# Plasma Concentrations of Matrilysins MMP-7 and MMP-26 as Diagnostic Biomarkers in Breast Cancer

**DOI:** 10.3390/jcm10071436

**Published:** 2021-04-01

**Authors:** Barbara Maria Piskór, Andrzej Przylipiak, Emilia Dąbrowska, Iwona Sidorkiewicz, Marek Niczyporuk, Maciej Szmitkowski, Sławomir Ławicki

**Affiliations:** 1 Department of Aesthetic Medicine, Medical University of Bialystok, 15-267 Bialystok, Poland; andrzej.przylipiak@umb.edu.pl (A.P.); emila_lubowicka@wp.pl (E.D.); niczy.ma@gmail.com (M.N.); 2 Clinical Research Centre, Medical University of Bialystok, 15-276 Bialystok, Poland; iwona.sidorkiewicz@umb.edu.pl; 3 Department of Biochemical Diagnostics, Medical University of Bialystok, 15-269 Bialystok, Poland; msz@umb.edu.pl; 4 Department of Population Medicine and Lifestyle Diseases Prevention, Medical University of Bialystok, 15-269 Bialystok, Poland; slawicki@umb.edu.pl

**Keywords:** breast cancer, matrilysins, metalloproteinases, MMP-7, MMP-26, plasma concentration

## Abstract

Metalloproteinases (MMPs) are a group of proteolytic enzymes involved in the maintenance of a proper structure of extracellular matrix (ECM). Matrilysins (MMP-7 and MMP-26) are members of the MMPs group that show promise as potential breast cancer (BC) markers. The aim of the study was to evaluate plasma levels of MMP-7, MMP-26 and CA 15-3 individually and in combination and assess the diagnostic utility of studied matrilysins in patients with BC. The study group consisted of 120 patients with BC, and the control group consisted of 40 subjects with benign breast cancer and 40 healthy women. Concentrations of MMP-7 and MMP-26 were determined by enzyme-linked immunosorbent assay, and CA 15-3 by chemiluminescent microparticle immunoassay. Plasma levels of MMP-7 were significantly higher in the BC group than in the control group. Concentrations of MMP-26 and CA 15-3 were highest in stages II and IV of the disease. The highest diagnostic sensitivity was observed in stages III and IV BC for the combination of all tested markers (92.5%). The highest diagnostic specificity was noted for all tested parameters combined in the BC group (95.0%). The area under the receiver operating characteristic (ROC) curve (AUC) for the combination of markers (MMP-7+MMP-26+CA 15-3) was the largest (0.9138) in stages III and IV. Individual marker analysis showed that MMP-7 had the highest AUC (0.8894) in advanced stages of the disease. Study results indicate that MMP-7 could be used as an additional marker that would improve the diagnostic utility of CA 15-3 in early stages of BC. Therefore, the combined assessment of MMP-7 and MMP-26 with CA 15-3 might be useful in determining disease progression. Further studies are needed to evaluate whether matrilysins show promise as potential markers for improving the diagnosis of BC.

## 1. Introduction

Breast cancer (BC) is the most commonly diagnosed cancer in females worldwide. It most frequently originates in the lactiferous ducts as a result of uncontrolled proliferation of epithelial cells [1]. Studies of pathological processes associated with tumour growth, and the occurrence of lymph node metastases and distant metastases reveal that matrix metalloproteinases (MMPs) are key proteins involved in shaping the tumour microenvironment and thus impacting cancer progression and metastases [2,3]. MMPs are a family of a proteolytic enzymes responsible for remodelling of the extracellular matrix (ECM). The majority of MMPs consist of a propeptide, a catalytic metalloproteinase domain, a linker peptide of variable lengths and a hemopexin (Hpx) domain. MMPs include matrilysins MMP-7 and MMP-26 which lack the linker peptide and the Hpx domain [4]. MMP-7 may affect the structure of casein, laminin, fibronectin, collagen III/IV/V/IX/X/XI, type I/II/IV/V gelatins, elastin and proteoglycans, thereby inducing their degradation [5]. Laminin, collagen IV and proteoglycans are the main components of the basement membrane [6]. Basement membrane degradation by MMPs plays a crucial role in local invasion to the surrounding stroma and in distant metastasis via blood and lymphatic vessels [7]. Transition of ductal carcinoma in situ to invasive ductal carcinoma, with higher mortality rates among patients, is associated with damage to the basement membrane [8]. The five–year survival rate for patients with BC in situ has been demonstrated to be 99%. It decreases to 85% for patients with metastases to the mammary gland tissue and falls to 27% for those with distant metastases [9]. MMP-7 regulates several biochemical processes such as the activation, degradation and shedding of non-ECM proteins. Heparin-binding epidermal growth factor precursor (proHB-EGF), membrane-bound Fas ligand (FasL), tumour necrosis factor (TNF) alpha precursor and E-cadherin are cleaved by MMP-7 into mature HB-EGF, soluble FasL, TNF-alpha and E-cadherin, which promote cellular proliferation and invasion [10]. MMP-7 is secreted by epithelial cells, macrophages, myolytic myocytes and tumour cells themselves [5,10,11,12,13]. What should be noted is that MMP-26 cleaves not only ECM components, e.g., vitronectin, fibrinogen, type IV collagen and gelatins, but also non-ECM proteins, such as insulin-like growth factor-binding protein-1 (IGFBP-1) and α-1 protease inhibitor [14,15]. Whereas the expression of MMP-26 in healthy tissues is decreased, it is enhanced in cancerous tissue of epithelial origin [16,17]. In physiological conditions, MMP-26 expression is detected in the sclera of the human eye and T-cells [18]. In women, MMP-26 expression is present in ovarian theca and granulosa cells and in various endometrial cells, e.g., surface and glandular epithelial cells, and vascular endothelial and endometrial stromal cells [19,20]. A study by Marchenko et al. demonstrated increased MMP-26 gene expression in various tumour cell lines including MCF-7 breast carcinoma cells. In addition, the authors indicated that MMP-26 is probably involved in the destruction of necrotic tissue of oxygen-deficient tumours and may participate in neovascularisation and angiogenesis [17].

It was proved that MMP-7 and MMP-26 can activate MMP-2 [21] and MMP-9 [14,21,22,23]. MMP-7 participates in the proteolytic cascade enabling the activation of the inactive form of MMP-9 (pro-MMP-9) to the active form. Moreover, MMP-7 may enhance the proteolytic activity of MMP-9, and also have an effect on a transient increase in the proteolytic activity of MMP-2 [21]. By activating MMP-9, MMP-26 may also cooperate with MMP-9 in the proteolytic cascade [14]. Our research team has been intensively studying the diagnostic usefulness of MMPs in gynaecological cancers, including MMP-2 and MMP-9 in breast cancer [24,25,26]. Available literature reports most commonly describe enhanced matrilysins expression in breast cancer cells, but few publications examine their plasma concentration in patients with breast cancer. Plasma matrilysins could be useful biomarkers in breast cancer diagnosis. Therefore, the aim of the present study was to determine plasma levels of MMP-7 and MMP-26 in comparison to the commonly accepted tumour marker (CA 15-3) in various stages of BC.

## 2. Materials and Methods

### 2.1. Study Participants

Table 1 shows study and control groups. The study group comprised 120 patients with BC referred to the Department of Oncology, Medical University of Bialystok, Poland, between 2015 and 2018. Classification and stage of the tumour were established according to the International Union against Cancer Tumour-Node-Metastasis (UICC-TNM) classification. Histopathology of breast cancer was evaluated in all cases by biopsy of mammary tumour tissue prior to or following surgery (all patients with ductal adenocarcinoma). Written consent and participants’ own statements regarding their medical history (i.e., a personal or family history of cancer, reproductive history, general health problems, hospitalisations, surgeries, medication use) and lifestyle habits, including smoking were obtained. None of the patients received chemotherapy or radiation therapy prior to blood sample collection. Initial assessment procedures included a physical examination and blood tests, mammography, a breast ultrasound, breast core biopsy and a chest X-ray. In addition, radioisotope bone scans, bone marrow aspiration and examination, and brain and chest tomography scans were performed if needed.

The control group consisted of 80 subjects divided into two groups: 40 patients with benign breast lesions and 40 healthy controls. All patients in the control group underwent a mammary gland examination performed by a gynaecologist and a breast ultrasound. The benign character of mammary lesions was confirmed by histopathological examination. In addition, women with inflammation and comorbidities such as circulatory disorders were excluded from the study.

The study was approved by the local Bioethics Committee (R-I-002/51/2015) and informed consent for study participation was obtained from all subjects.

### 2.2. Plasma Collection and Storage

Venous blood samples were obtained from all study participants. Blood was collected into EDTA tubes (S-Monovette, Sarstedt, Germany). Plasma represents the optimal choice anticoagulants to better evaluate MMPs for clinical and diagnostic purposes. There is strong evidence that serum should not be used to assess circulating MMP levels in clinical studies. The significant differences in MMP concentrations measured in plasma versus serum are attributable to the release of these biomarkers by platelets and leukocytes during the clotting process in the serum tube, however the use of an anticoagulant in the collected blood prevents this artefact [27,28,29]. Plasma samples were obtained by centrifugation at 1000× *g* for 15 min at 2–8 °C and stored at −85 °C until assayed.

### 2.3. Measurement of MMP-7, MMP-26 and CA 15-3

The tested parameters (MMP-7 and MMP-26) were measured with enzyme-linked immunosorbent assay (ELISA) (MMP-7—R&D systems, Abingdon, UK; MMP-26—EIAab Science, Wuhan, China), according to the manufacturer’s instructions. Plasma concentrations of CA 15-3 were measured by chemiluminescent microparticle immunoassay (CMIA) (Abbott, Chicago, IL, USA). The intra and inter-assay coefficient were checked by the manufacturers of the diagnostic kits to comply with standards.

### 2.4. Statistical Analysis

Statistical analysis was performed using the STATISTICA 12.0 program (StatSoft, Tulsa, OK, USA). The Shapiro–Wilk test showed that the obtained data did not follow a normal distribution. Therefore, the Mann–Whitney U test, the Kruskal–Wallis test and multivariate analysis of various data by the post hoc Dwass–Steel–Critchlow–Fligner test were used to determine differences between the groups. Statistical significance was determined at the *p* < 0.05 level. Diagnostic sensitivity (SE), diagnostic specificity (SP), predictive value of a positive test result (PPV) and predictive value of a negative test result (NPV) were calculated according to standards described in the scientific literature [30]. The cut-off values were based on the 95th percentile. The cut-off values of CA 15-3, MMP-7 and MMP-26 (23.27 U/mL, 3.57 ng/mL and 13.06 ng/mL, respectively) were used at a specificity higher than 95% (calculated from healthy blood donors). Comparison of the diagnostic power of all studied markers was assessed using the areas under the receiver operating characteristic (ROC) curves (AUC) created using the GraphRoc program for Windows (Windows, Royal, AR, USA). Healthy participants and benign breast tumour subjects constituted the control group in analyses of diagnostic performance (SE, SP) and ROC curves.

## 3. Results

**Figure 1:**
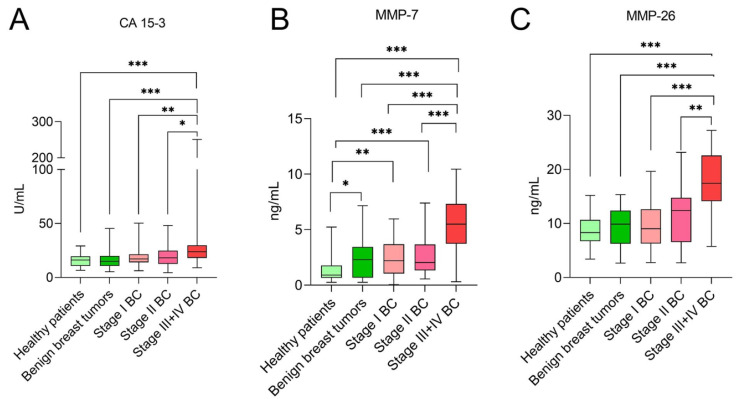
Plasma levels of tested parameters: CA 15-3 (**A**), MMP-7 (**B**) and MMP-26 (**C**) in patients with BC, benign breast tumour and healthy patients. Asterisks indicate significant differences (*, *p* < 0.05; **, *p* < 0.01; ***, *p* < 0.001). BC, breast cancer; CA 15-3, cancer antigen 15-3; MMP-7, matrix metalloproteinase 7; MMP-26, matrix metalloproteinase 26.

**Figure 2:**
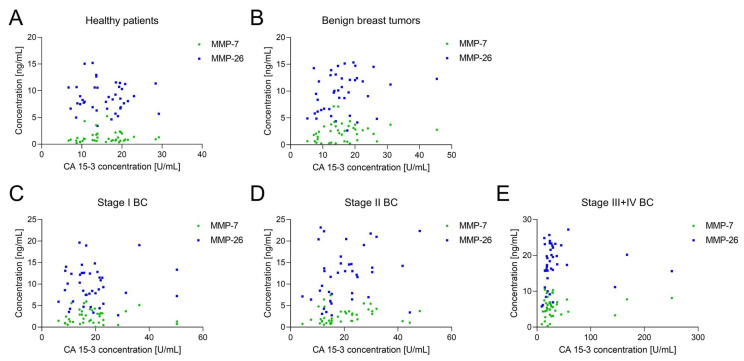
Scatterplots for plasma levels of MMP-7 and MMP-26 from CA 15-3 in healthy (**A**), benign breast tumour (**B**) and BC patients: Stage I (**C**), Stage II (**D**), Stage III + IV (**E**). BC, breast cancer; CA 15-3, cancer antigen 15-3; MMP-7, matrix metalloproteinase 7; MMP-26, matrix metalloproteinase 26.

Figure 1 presents plasma levels of tested parameters in patients with BC, benign breast tumour and healthy patients. Scatter plots were performed to present the relationships between MMPs and routinely used CA 15-3 in study groups (Figure 2). Patients with BC displayed significantly higher concentrations of MMP-7, MMP-26 and CA 15-3 in comparison to healthy subjects (MMP-7 and MMP-26 *p* < 0.001; CA 15-3 *p* = 0.002). Moreover, median levels of MMP-7 in all BC stages were higher than in healthy controls (stage I *p* = 0.003; stages II, III and IV *p* < 0.001). Interestingly, only in stages III and IV were median levels of MMP-26 and CA 15-3 significantly higher in comparison to healthy subjects (*p* < 0.001).

When patients with BC were compared with benign breast tumour subjects, a similar relationship was observed. Median levels of MMP-7, MMP-26 and CA 15-3 in the total BC group were higher than in the benign breast tumour group (*p* = 0.013; *p* = 0.015; *p* = 0.003, respectively). Furthermore, concentrations of all tested parameters were significantly higher in patients with stage III and IV BC in comparison to benign breast tumour subjects (*p* < 0.001), in whom, however, the concentration of MMP-7 was higher than in healthy participants (*p* = 0.03).

The concentrations of the tested parameters in patients with BC were tumour stage- dependent. Median levels of MMP-7, MMP-26 and CA 15-3 in stages III and IV were significantly higher in comparison to stage I (for MMPs *p* < 0.001; for CA 15-3 *p* = 0.003) and stage II (*p* < 0.001; *p* = 0.001; CA 15-3 *p* = 0.043, respectively).

The concentrations of MMP-7, MMP-26 and CA 15-3 in the BC group were significantly higher than in the total control group (benign breast tumour subjects and healthy controls) (*p* < 0.001). Median levels of all tested parameters in stages III and IV were enhanced in comparison to the control group (*p* < 0.001). Moreover, MMP-7 concentrations in stage II were statistically higher than in the control group (*p* = 0.013).

Table 2 presents diagnostic criteria: sensitivity (SE), specificity (SP), predictive value of a positive test result (PPV) and predictive value of a negative test result (NPV) in patients with BC. The SE of MMP-7 and MMP-26 in the total BC group was the same for both enzymes (45.0%) and was also higher when compared to CA 15-3. The highest SE was observed for the combination of all investigated markers (MMP-7+MMP-26+CA 15-3) (63.6%).

The SE of all tested parameters increased with cancer stage. In stage I, SE was highest for MMP-7 (27.5%). Interestingly, in stage II, the highest SE was observed for MMP-26 (37.5%). Furthermore, in stages III and IV, higher SE was found for MMPs (for MMP-7 75.0%; for MMP-26 77.5%) than for CA 15-3 (57.5%). Moreover, increasing SE was observed in every stage of cancer for the combination of markers: MMP-7 + CA 15-3 (stage I: 35.0%; stage II: 45.0%; stages III + IV: 90.0%); MMP-26 + CA 15-3 (stage I: 27.5%; stage II: 50.0%; stages III + IV: 87.5%); MMP-7 + MMP-26 + CA 15-3 (stage I: 40.0%; stage II: 57.5%; stages III + IV: 92.5%).

The diagnostic SP of all tested parameters was very high in the total group of cancer patients and in all stages of cancer (95.0%). SP for the combination of MMPs with CA 15-3 was lower (85.0%) than for the combinations of MMP-7 + CA 15-3 and MMP-26 + CA 15-3, where SP values were the same in both cases (90.0%).

Among the tested parameters, the predictive value of a positive test result (PPV) in the group of patients with BC was marginally higher for MMPs (96.4% for MMP-7 and MMP-26) than for CA 15-3 (95.1%). PPV in stage I was highest for MMP-7 (84.6%), but in stage II it was highest for MMP-26 (88.2%). In stages III and IV, PPV was similar for both enzymes (MMP-7: 93.7%; MMP-26: 93.9%). Among all tested markers, PPV increased with cancer stage. The combined assessment of the tested parameters and CA15-3 resulted in an increase in PPV in every stage of the tumour.

The predictive value of a negative test result (NPV) in the BC group was higher for MMPs in comparison to CA 15-3 (36.5% and 31.9%, respectively). NPV was highest for all tested parameters in stages II and IV (MMP-7: 79.2%; MMP-26: 80.9%; CA 15-3: 69.1%). In stage I, the highest NPV value was found for MMP-7 (56.7%), in stage II—MMP-26 (60.3%). The combined assessment of the tested parameters and CA15-3 resulted in an increase in NPV in every stage of the tumour. However, the highest NPV values were observed in stages III and IV (MMP-7 + CA 15-3: 90.0%; MMP-26 + CA 15-3: 87.8%; MMP-7 + MMP-26 + CA 15-3: 87.2%).

The relationship between the diagnostic SE and SP is illustrated by the ROC curve in Table 3. The AUC indicates potential clinical usefulness of a tumour marker and therefore its diagnostic power.

**Figure 3:**
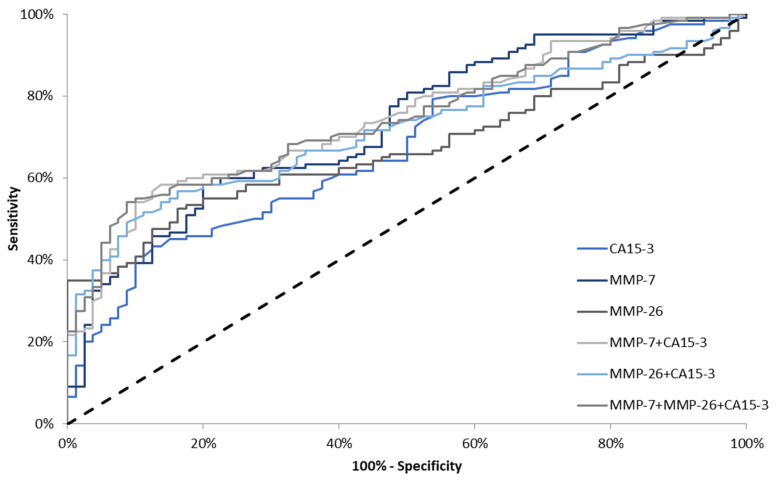
Diagnostic criteria of ROC curve in total breast cancer (BC) group. CA 15-3—cancer antigen 15-3; MMP-7—matrilysin-1; MMP-26—matrilysin-2.

In the total group of BC, the AUCs for all parameters were significantly higher in comparison to AUC = 0.5 (*p* < 0.001). The AUC for MMP-7 (0.7306) in the total BC group was larger than for MMP-26 (0.6720) and CA 15-3 (0.6743). Using a combination of markers, e.g., CA 15-3 and MMP-7 or MMP-26 resulted in an increase in AUC (0.7464; 0.7157, respectively). We observed that the AUC for the combination of CA 15-3 and MMP-7 was marginally larger than that for the combination of CA15-3 with MMP-7 and MMP-26 (Figure 3).

**Figure 4:**
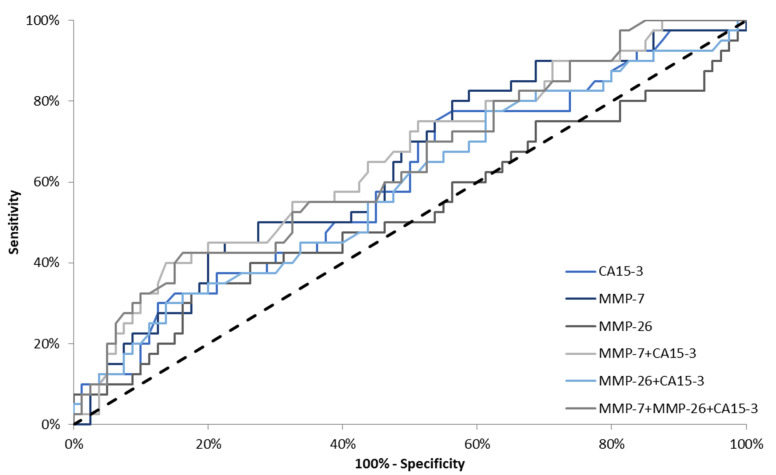
Diagnostic criteria of ROC curve in stage I breast cancer (BC). CA 15-3—cancer antigen 15-3; MMP-7—matrilysin-1; MMP-26—matrilysin-2.

In stage I BC, the highest AUC value was observed for the combination of CA 15-3 with MMP-7 (0.6538; *p* = 0.004)—the value was higher than that found for the combination of CA 15-3, MMP7 and MMP-26 (0.6413; *p* = 0.009). Considering single markers, the highest AUC was observed for MMP-7 (0.6328; *p* = 0.0136) (Figure 4).

**Figure 5:**
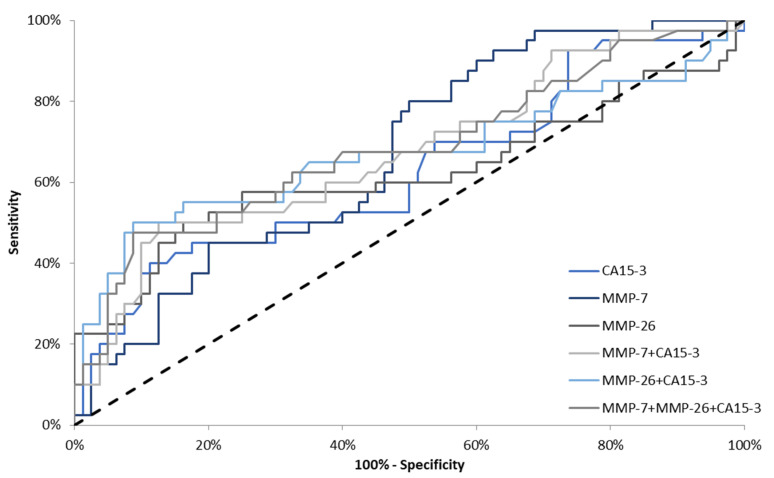
Diagnostic criteria of ROC curve in stage II breast cancer (BC). CA 15-3—cancer antigen 15-3; MMP-7—matrilysin-1; MMP-26—matrilysin-2.

**Figure 6:**
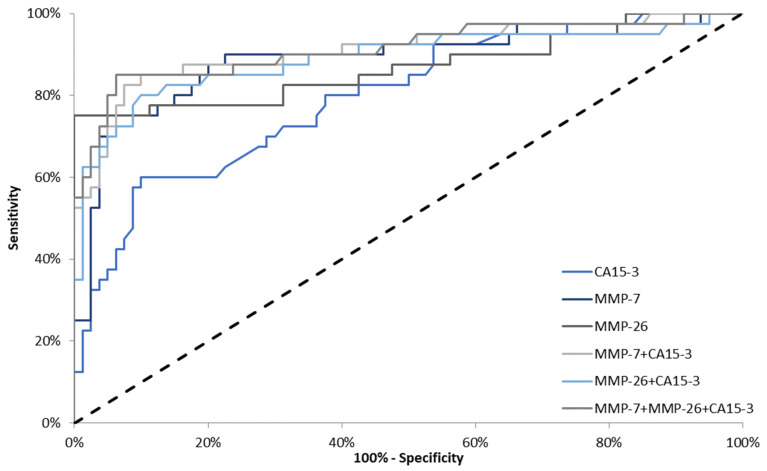
Diagnostic criteria of ROC curve in stages III and IV breast cancer (BC). CA 15-3—cancer antigen 15-3; MMP-7—matrilysin-1; MMP-26—matrilysin-2.

In stage II BC, the highest AUC was observed for the combination of all tested parameters (0.6834; *p* = 0.0009). Marginal differences in areas under the ROC curves were observed for the combination of CA 15-3 with MMP-7 and MMP-26 (0.6745; *p* = 0.0013 and 0.6711; *p* = 0043, respectively). Regarding all tested parameters, the highest AUC was presented by MMP-7 (0.6697; *p* = 0.0007). For MMP-26 and CA 15-3, AUC values were very similar (0.6216; *p* = 0.0499 and 0.6270; *p* = 0.0252, respectively) (Figure 5). In stages III and IV BC, the AUC for MMP-7 (0.8894) was larger than the AUCs for MMP-26 (0.8684) and CA 15-3 (0.7970). We also observed that the AUC for the combination of all studied parameters (0.9138) was higher in comparison to the combination of CA 15-3 with MMP-7 and MMP-26 (0.9109; 0.8905, respectively). The AUCs for all parameters were significantly larger in comparison to AUC = 0.5 (*p* < 0.001 in all cases) (Figure 6).

## 4. Discussion

MMPs play a significant role in BC progression. They participate in the modulation of the immune system, angiogenesis and development of the tumour microenvironment, which facilitates cancer progression. Their ability to disintegrate ECM components is considered a key factor leading to disease development [31]. To date, the most extensive research efforts have focused on MMP-2 and MMP-9, which are thought to have a significant impact on the development of BC [25,26,32]. Nevertheless, determining the functions of other MMPs is crucial since it may help elucidate their role in BC progression. In the present paper we focused on plasma levels of MMP-7 and MMP-26 in patients with BC. In addition, we compared the tested enzymes with CA 15-3, the protein commonly determined in patients with BC, and evaluated whether combining the enzymes with each other or with CA 15-3 demonstrated a promising diagnostic value. To the best of our knowledge, our research team is the first to analyse plasma concentrations of MMP-7 and MMP-26 in combination with CA 15-3 in patients with BC. However, the role of circulating levels of MMP-7 and MMP-26 in cancer progression and development has still not been elucidated.

In 2006, the American Society of Clinical Oncology (ASCO) endorsed that in the absence of specific clinical exam findings, testing serum biomarkers, including carcinoembryonic antigen (CEA), cancer antigen (CA) 15-3 and CA 27-29, is not recommended. Rising tumour markers are concerning for tumour progression but may also be seen in the setting of responding disease. An isolated increase in tumour markers should rarely be used to declare progression of disease. However, serum tumour markers such as CA 15-3 and CEA are the most widely used serum tumour markers for surveillance purposes and treatment response in clinical practice. One study has found a strong association between tumour marker velocity and breast cancer recurrence. Tumour marker velocity may be a useful adjunct to absolute tumour marker values to distinguish between clinically significant elevated tumour markers from baseline variation. This suggests the clinical utility of serial CA 15-3 and CEA measurements in breast cancer surveillance [33,34]. Serum tumour markers such as CA 15-3 and CEA are still the most widely used serum tumour markers for surveillance purposes and treatment response in clinical practice. Although the ASCO panel does not recommend therapeutic decisions be based on the serum tumour marker status, several studies showed that the preoperative concentration of tumour markers could be useful when deciding on treatment strategy [35,36,37].

The present study revealed that patients with BC had significantly higher plasma concentrations of MMP-7, MMP-26 and CA 15-3 than healthy controls. Moreover, MMP-7 concentrations were enhanced in subjects with benign breast tumour in comparison to healthy controls. We hypothesised that plasma concentrations of the studied parameters may be predictive factors when distinguishing between healthy individuals and subjects with BC or even benign breast lesions. Consequently, patients with stage III and IV BC had significantly higher MMP-7, MMP-26 and CA 15-3 levels in comparison to patients with stage I BC and the control group. There are no reports in the available literature regarding plasma concentrations of matrilysins in patients with BC. Clinical studies suggest that circulating MMPs may constitute an early sign of BC [38,39]. Nevertheless, case-control studies, regarding the relationship between the plasma concentration of MMPs, including MMP-7 and the subsequent risk of postmenopausal breast cancer have not demonstrated any dependence. No differences in concentrations between the study group and the control group were observed. Nor were any differences found between the concentration of MMPs and the occurrence of mammary cancer [39]. Consistent with the data cited above is a study by Aroner et al. [40]. They also performed a 10-year follow-up study investigating plasma MMPs (e.g., MMP-7) and BC risk. The authors did not find any significant associations between the investigated MMPs and BC subtypes, although a positive correlation between MMP7 concentrations and node metastases was suggested. This indicated that MMP-7 is not a suitable marker for detection of early stages of BC. However, it appears to be an appropriate marker in the diagnosis and monitoring of treatment response in advanced stages of BC [40].

Katunina et al. performed analysis of MMPs, including MMP-7 in the tumour tissue, adjacent histologically intact tissue and serum of patients with BC [41]. An enzyme immunoassay test revealed higher MMP-7 levels in cancer tissue in comparison to healthy tissue. The authors did not establish any correlations between MMP-7 concentrations in tissue and serum. Serum analysis did not show significantly elevated MMP-7 concentrations in patients with BC compared to controls [41]. The results of the study are contradictory to our observations. Nevertheless, it is worth noting that the authors used serum not plasma, as in the present investigation, in their study. In addition, the study was conducted using a small study and control group—45 women with breast cancer and 8 healthy controls. Therefore, the small study sample may be the reason for the authors’ inability to establish any significant relationships between the tested groups.

It is known that MMP-26 is involved in the development of oestrogen-dependent cancers, including breast cancer [42,43]. BC cells expressing MMP-26 are characterised by an increased number of mitotic figures, atypia, presence of glycogen fields and atypical lysosomes in the cytoplasm. Yang et al. demonstrated that the ability of these cells to migrate was significantly enhanced when compared to the control group. However, the presence of anti-MMP-26 antibodies impaired it substantially. The number and length of blood vessels produced as a result of induction by cells expressing MMP-26 was higher than those induced by tumour cells not expressing MMP-26. Expression of MMP-26 increased the malignant phenotype of these cells in vivo [43]. However, literature lacks reports regarding plasma levels of MMP-26 in BC patients. We found data on plasma levels of both matrilysins in a paper by Galewska et al. The authors evaluated concentrations of MMP-7 and MMP-26 in the plasma and serum of umbilical cord blood [44]. However, since the study is not related to breast cancer and there are no other reports on the subject in the available literature, we were unable to compare our findings regarding MMP-26 or both enzymes with the research of other authors.

Sensitivity, specificity and area under the ROC curve characterise the diagnostic usefulness of tumour markers. In the present study, higher values of SE for all tested parameters were observed. SP for individual matrilysin and CA 15-3 was the same in all studied groups (95%). Similar results were obtained for MMP-7 by Będkowska et al. who analysed MMP-7 concentrations in epithelial ovarian cancer [45]. The authors observed higher concentrations of MMP-7 in epithelial ovarian cancer patients in comparison to healthy controls. Moreover, they revealed that SE values increased with tumour progression. SP was the same in all stages of the disease (95%). As for AUC values, they showed significantly higher AUCs when compared to AUC = 0.5 in all studied ovarian cancer groups [45]. In our study, the AUCs for all investigated markers were significantly higher compared to AUC = 0.5 in stages II, III and IV cancer. In stage I BC, only single analysis of MMP-7 or in conjunction with MMP-26 and CA 15-3 demonstrated diagnostic utility. A study by Leelawat et al. described the diagnostic utility of MMP-7 in cholangiocarcinoma [46]. The authors observed enhanced MMP-7 concentrations in the serum of patients with cholangiocarcinoma. SE and SP values were higher for the matrilysin than for CA 19-9, a marker commonly used in the diagnosis of gastrointestinal cancers. Interestingly, AUC analysis showed that MMP-7 was more accurate than CA 19-9 in diagnosing cholangiocarcinoma [46]. Vocka et al. studied the accuracy of MMP-7 in diagnosing metastatic colorectal cancer [47]. The authors compared serum levels of MMP-7 with CEA and CA 19-9. The concentration of MMP-7 was significantly elevated in patients with colorectal cancer compared to healthy controls. MMP-7 had very similar SE and SP as CEA, but its SE was superior to CA 19-9. Serum levels of MMP-7 correlated with worse outcomes and had a prognostic value [47]. The results presented above regard different types of cancer than BC, but reveal a similar trend, i.e., elevated levels of MMP-7 in cancer patients, high values of SE and SP and higher values of AUC compared to AUC = 0.5. Unfortunately, we could not compare our data regarding MMP-26 or the combination of MMP-7 and MMP-26 with each other, and with CA 15-3 since no reports on the subject are available.

Our study has some limitations. Oestrogen receptor (ER) status was not included in clinical characteristics of studied patients. Thus, no association study between MMPs and ER expression was performed. Nevertheless, the research is innovative as the present paper is the first report regarding the diagnostic usefulness of the set of markers MMP-7 and MMP-26 in combination with CA 15-3 in the diagnosis of BC.

## 5. Conclusions

Our results indicate that MMP-7 and MMP-26 are promising markers in the diagnosis of BC. Furthermore, the results presented in the paper indicate that the combined analysis of MMP-7 and MMP-26 with CA 15-3 may be useful in determining disease progression. Moreover, MMP-7 may be introduced as a breast tumour biomarker, particularly in the diagnosis of early-stage BC.

## Figures and Tables

**Table 1:** Characteristics of examined groups: breast cancer (BC) patients and control groups.

Study Group			Number of Cases
Study groups: breast cancer patients	Ductal adenocarcinoma		120
	Median age (range)		54 (21–85)
	Tumour stage	I	40
		II	40
		III + IV	40
	Menopausal status		
	Premenopausal		38
	Postmenopausal		82
Control groups	Benign breast tumour lesions subjects		40
	adenoma fibroadenoma		18
			22
	Median age (range)		44 (33–63)
	Menopausal status		
	Premenopausal		17
	Postmenopausal		23
	Healthy women		40
	Median age (range)		46 (25–64)
	Menopausal status		
	Premenopausal		18
	Postmenopausal		22

**Table 2:** Diagnostic criteria of tested parameters in patients with BC.

Tested Parameters	Diagnostic Criteria (%)	Breast Cancer			
		Stage I	Stage II	Stages III + IV	Total Group
MMP-7	SE	27.5	32.5	75.0	45.0
	SP	95.0	95.0	95.0	95.0
	PPV	84.6	86.7	93.7	96.4
	NPV	56.7	58.5	79.2	36.5
MMP-26	SE	20.0	37.5	77.5	45.0
	SP	95.0	95.0	95.0	95.0
	PPV	80.0	88.2	93.9	96.4
	NPV	54.3	60.3	80.9	36.5
CA 15-3	SE	12.5	27.5	57.5	32.5
	SP	95.0	95.0	95.0	95.0
	PPV	71.4	84.6	92.0	95.1
	NPV	52.1	56.7	69.1	31.9
MMP-7 + CA 15-3	SE	35.0	45.0	90.0	56.7
	SP	90.0	90.0	90.0	90.0
	PPV	77.8	81.8	92.3	94.4
	NPV	58.1	62.1	90.0	40.9
MMP-26 + CA 15-3	SE	27.5	50.0	87.5	55.0
	SP	90.0	90.0	90.0	90.0
	PPV	73.3	83.3	89.7	94.3
	NPV	55.4	64.3	87.8	40.0
MMP-7 + MMP-26 + CA 15-3	SE	40.0	57.5	92.5	63.3
	SP	85.0	85.0	85.0	85.0
	PPV	72.7	79.3	86.1	92.7
	NPV	58.6	66.7	87.2	43.6

BC—breast cancer; MMP-7—matrilysin-1; MMP-26—matrilysin-2; CA 15-3—cancer antigen 15-3; SE—diagnostic sensitivity; SP—diagnostic specificity; PPV—predictive value of a positive test result; NPV—predictive value of a negative test result.

**Table 3:** Diagnostic criteria of ROC curve for tested parameters in BC.

Tested Parameters	AUC	SE	95% C.I. (AUC)	*p* (AUC = 0.5)
ROC Criteria in Breast Cancer (Total Group)				
MMP-7	0.7306	0.0355	(0.661–0.800)	<0.001
MMP-26	0.6720	0.0375	(0.599–0.745)	<0.001
CA 15-3	0.6743	0.0378	(0.600–0.748)	<0.001
MMP-7 + CA 15-3	0.7464	0.0341	(0.680–0.813)	<0.001
MMP-26 + CA 15-3	0.7157	0.0357	(0.646–0.786)	<0.001
MMP-7 + MMP-26 + CA 15-3	0.7461	0.0339	(0.680–0.813)	<0.001
ROC Criteria in Breast Cancer (Stage I)				
MMP-7	0.6328	0.0538	(0.527–0.738)	0.0136
MMP-26	0.5259	0.0597	(0.409–0.643)	0.6638
CA 15-3	0.5988	0.0556	(0.490–0.708)	0.0755
MMP-7 + CA 15-3	0.6538	0.0535	(0.549–0.759)	0.0040
MMP-26 + CA 15-3	0.5856	0.0565	(0.475–0.696)	0.1297
MMP-7 + MMP-26 + CA 15-3	0.6413	0.0540	(0.535–0.747)	0.0090
ROC Criteria in Breast Cancer (Stage II)				
MMP-7	0.6697	0.0500	(0.572–0.768)	0.0007
MMP-26	0.6216	0.0620	(0.500–0.743)	0.0499
CA 15-3	0.6270	0.0568	(0.516–0.738)	0.0252
MMP-7 + CA 15-3	0.6745	0.0541	(0.568–0.781)	0.0013
MMP-26 + CA 15-3	0.6711	0.0599	(0.554–0.788)	0.0043
MMP-7 + MMP-26 + CA 15-3	0.6834	0.0551	(0.575–0.791)	0.0009
ROC Criteria in Breast Cancer (Stages III + IV)				
MMP-7	0.8894	0.0361	(0.819–0.960)	<0.001
MMP-26	0.8684	0.0419	(0.786–0.951)	<0.001
CA 15-3	0.7970	0.0433	(0.712–0.882)	<0.001
MMP-7 + CA 15-3	0.9109	0.0320	(0.848–0.974)	<0.001
MMP-26 + CA 15-3	0.8905	0.0375	(0.817–0.964)	<0.001
MMP-7 + MMP-26 + CA 15-3	0.9138	0.0325	(0.850–0.977)	<0.001

BC—breast cancer; MMP-7—matrilysin-1; MMP-26—matrilysin-2; CA 15-3—cancer antigen 15-3; ROC—the receiver operating characteristic; AUC—the area under the curve; SE—sensitivity; 95% C.I.—95% confidence interval.

## Data Availability

The datasets used and analysed during the current study are available from the corresponding author on reasonable request.
